# Investigation into the Performance of TDR and FDR Techniques for Measuring the Water Content of Biochar-Amended Loess

**DOI:** 10.3390/s25133970

**Published:** 2025-06-26

**Authors:** Nan Zhou, Ziyi Zhao, Ming Li, Junping Ren, Ping Li, Qiang Su

**Affiliations:** 1 College of Civil Engineering and Mechanics, Lanzhou University, Lanzhou 730000, China; zhoun2023@lzu.edu.cn (N.Z.); zhaoziyi2024@lzu.edu.cn (Z.Z.); liming2024@lzu.edu.cn (M.L.); 2 State Key Laboratory of Continental Dynamics, Department of Geology, Northwest University, Xi’an 710069, China; liping_dzxx@nwu.edu.cn; 3 China National Geological & Mining Corporation, Beijing 100029, China; suqiang@chinagm.com.cn

**Keywords:** soil water content, biochar amendment, time domain reflectometry, frequency domain reflectometry, dielectric constant, calibration equations

## Abstract

**Highlights:**

What are the main findings?

- Biochar significantly affects the accuracy of TDR and FDR measurements.
- Water content measurement accuracy is related to biochar dosage and particle size.
- Dielectric mixing model can well describe the dielectric constant of BAS.
- Calibration equations are more accurate than empirical equations for BAS.

What is the implication of the main finding?

- It can promote the application of TDR and FDR in measuring the water content of BAS.
- The dielectric mixing model can provide a theoretical basis for calculating the dielectric constant of BAS.
- Calibration can improve the accuracy of water content measurement in biochar-amended soil.

**Abstract:**

Biochar has garnered considerable attention for its potential to improve soil properties due to its unique characteristics. However, the precise measurement of soil water content using electromagnetic sensors becomes challenging after biochar is incorporated. This study investigated the impact of biochar on soil water content measurement by adding biochar of varying dosages and particle sizes to a typical loess, under both room and subzero temperature conditions by using time domain reflectometry (TDR) and frequency domain reflectometry (FDR) techniques. The results demonstrate that biochar amendment significantly influenced the measurement accuracy of both TDR and FDR. A clear dosage-dependent relationship was observed, with measurement errors exhibiting progressive escalation as biochar addition rates increased. At room temperature, the root mean square error (RMSE) values for loess were remarkably low (TDR: 0.029; FDR: 0.093). In contrast, the 9% coarse-grained biochar-amended soil (BAS-9%C) showed substantially elevated RMSE values (TDR: 0.2006; FDR: 0.1468). Furthermore, comparative analysis revealed that particle size significantly affected measurement precision, with coarse-grained biochar demonstrating more pronounced interference effects than fine-grained biochar at equivalent application rates. At subzero temperatures, BAS-6%C exhibited significantly higher RMSE values (TDR: 0.1753; FDR: 0.2022) compared to BAS-6%F (TDR: 0.079; FDR: 0.1872). A dielectric mixing model was established for calculating the dielectric constant of BAS. In addition, calibration equations for accurately determining the water content of biochar-amended loess under both room and subzero temperature conditions were established. Furthermore, the mechanisms by which biochar influenced the performance of the TDR and FDR sensors are comprehensively discussed. These findings can provide valuable theoretical foundation and practical guidance for future soil improvement with biochar and accurate water content measurement in BAS.

## 1. Introduction

Soil water content represents a fundamental parameter that determines soil physical properties, directly influencing the water retention capacity, aeration, hydraulic conductivity, and thermodynamic characteristics [1,2]. Consequently, accurate monitoring of soil moisture has long been recognized as a crucial research focus [3]. In agricultural production, accurate soil moisture measurement facilitates the development of smart agriculture by reducing water resource wastage while maintaining stable and high crop yields [4,5,6]. In geoenvironmental engineering, water status significantly affects soil mechanical behaviors, including shear strength, compressibility, slope stability, and foundation bearing capacity [7,8]. Particularly in permafrost regions or seasonal freeze–thaw areas, phase transitions of soil water can substantially modify soil engineering properties [9]. Therefore, accurate assessment of unfrozen water content is not only critical for understanding the hydrothermal coupling processes but also provides essential scientific guidance for infrastructure construction in cold regions [10,11].

Among the various soil water measurement methods, time domain reflectometry (TDR), frequency domain reflectometry (FDR), and nuclear magnetic resonance (NMR) are the most commonly used techniques. TDR determines water content by measuring the dielectric constant of the bulk soil sample, which can be inferred from the reflection coefficient of the electromagnetic waves transmitted along the TDR probe. TDR offers advantages such as fast measurement, cost-effectiveness and portability, making it a widely used non-destructive and automated method [12,13]. FDR is similar to TDR in that it also measures the soil dielectric constant to determine the water content, but it utilizes the frequency response for that purpose. FDR moisture sensors interact with soil using electromagnetic waves at specific frequencies to calculate the soil dielectric constant. NMR, on the other hand, determines the water content by measuring the spatial density of hydrogen atoms in a soil sample. This method uses a magnetic field to align hydrogen atoms, followed by a radiofrequency pulse that excites them to a higher energy state. When the radiofrequency pulse stops, the atoms relax back, emitting detectable signals. By analyzing these signals, the hydrogen density can be determined and soil water content can be further obtained [14].

Climate change is the most significant environmental issue humanity is facing to date and one of the major challenges of the 21st century [15]. The substantial emissions of greenhouse gases have intensified the global greenhouse effect, leading to a series of disasters, such as increased frequency of extreme weather events, rising sea levels, and glacial melting [16,17]. In recent years, biochar has garnered widespread attention as a low-cost carbon sequestration technology, which can sequester large amounts of CO_2_ through the biomass carbonization process, thereby mitigating the greenhouse effect [18,19]. Specifically, biochar can be produced from a variety of biomass materials under high-temperature, oxygen-limited conditions [20,21], and it typically possesses a high specific surface area and a rich array of surface functional groups [22,23]. These characteristics enable biochar to effectively adsorb and retain substantial amounts of water and nutrients [24,25,26]. Consequently, biochar is regarded as an excellent soil additive capable of improving the physical and chemical properties of soils [27,28].

As a soil amendment material, biochar’s roles in enhancing soil structure and improving water retention capacity have been widely studied [29]. However, the impact of biochar addition on the accuracy of soil water measurement has been less explored and remains a topic worthy of in-depth investigation, particularly when using common techniques such as TDR and FDR. As mentioned above, TDR and FDR probes measure soil water content based on its dielectric constant, but the dielectric properties of biochar differ from those of the soil constituents, which may lead to measurement discrepancies [30]. Several studies have indicated that the biochar type and amount added and the type of soil significantly influence the measurement accuracy of TDR and FDR [31,32]. Therefore, when biochar is added to soils, it is essential to calibrate the TDR and FDR results to ensure the accuracy of water content measurement. To this end, studies have explored soil water measurement models under different organic matter treatments and have proposed optimization strategies to mitigate the negative effects of organic matter on measurement accuracy [33,34,35].

To close the above knowledge gaps, the present study investigated the effects of biochar on the accuracy of soil water content measurement by using TDR and FDR techniques, under both room temperature and subzero temperature conditions. A typical loess soil and a corn straw biochar were used for this purpose. A series of compacted biochar-amended loess (BAS hereafter) was prepared with varying biochar dosages and particle sizes, and their water contents were subsequently determined and analyzed. The effects of biochar dosage and particle size on the electromagnetic waves of the TDR probe, and on the electrical conductivity and dielectric constant of the BAS specimens were also studied. In addition, specific calibration curves were established for the water content of the BAS specimens under room temperature and subzero temperatures. The characteristics of the pore structure of the amended specimens were also revealed by NMR. Finally, the mechanisms by which biochar influences the performance of commonly used TDR and FDR sensors were discussed. The results of the present study not only hold theoretical significance but also offer practical guidance for accurately determining the water content of BAS for soil science and engineering practices.

## 2. Experimental Materials and Methods

### 2.1. Experimental Materials

The loess samples were collected from the northern part of the Jiuzhoutai area (36°6′36″ N, 103°47′38.4″ E) of Lanzhou, Gansu province, China. The collected samples were air-dried and sieved through a 2 mm standard sieve. The specific gravity, Atterberg limits, and compaction characteristics of the loess were obtained following ASTM designations [36,37,38]. The pH of the loess was determined by using a portable pH tester. The biochar used is available from a company in Liaoning province, China, and is produced from corn straw through a pyrolysis process under oxygen-limited conditions at temperatures ranging from 400 °C to 600 °C. Measurements of the specific gravity, ash content, pH and surface ion content of the biochar were conducted. These basic properties of the loess and biochar are summarized in Table 1.

**Figure 1:**
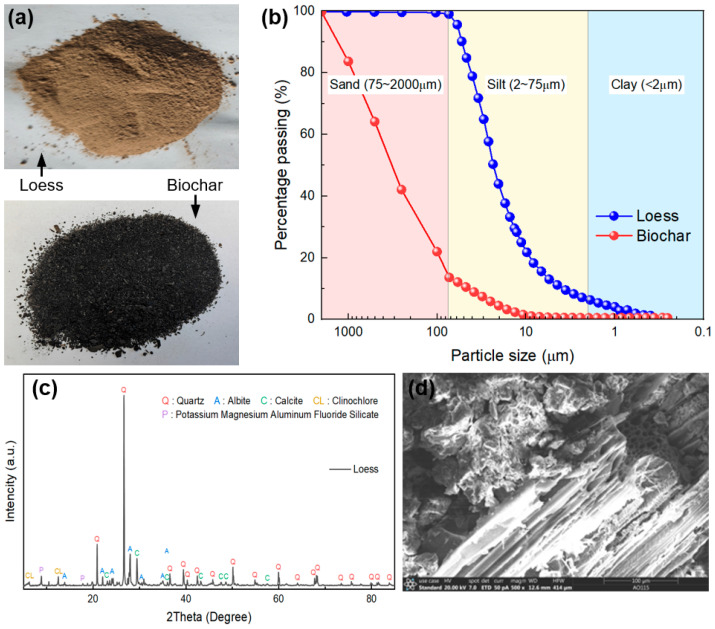
(**a**) Photos and (**b**) particle size distributions of loess and biochar, (**c**) XRD pattern of loess, and (**d**) SEM of loess amended with 9% coarse-grained biochar.

Figure 1a,b present the photographs of loess and biochar, along with their particle size distributions. Particle size analysis was conducted via mechanical sieving for particles larger than 0.075 mm, and via a laser diffraction particle size analyzer for smaller particles (<0.075 mm). It can be seen that the tested loess was light yellow in color, with a relatively uniform particle size distribution concentrated in the range of 0.002 to 0.075 mm, predominantly consisting of silt-sized particles. In contrast, the biochar particles were naturally darker and coarse-grained, with most particle sizes exceeding 0.075 mm. The X-ray diffraction (XRD) pattern of the loess is shown in Figure 1c, revealing that the main mineral components of the loess were quartz, albite, and calcite. The Fourier transform infrared spectroscopy (FTIR) spectrum of the biochar demonstrates that various functional groups, such as –OH, –CH, C–O, C=C, and –CH_3_, existed on the surface of the biochar. Figure 1d displays the scanning electron microscopy (SEM) image of the loess amended with 9% coarse-grained biochar (i.e., 0.075~2 mm, see Section 2.3) as an example. The image illustrates that the coarse-grained biochar possessed a rich porous structure, which may have complex physical interactions with soil constituents along with its chemical effects, contributing to its effectiveness as a soil amendment.

### 2.2. Basic Principles of TDR, FDR, and NMR

#### 2.2.1. Time Domain Reflectometry (TDR)

TDR is a method that measures the time taken for electromagnetic waves to propagate through a probe inserted into a soil, utilizing a TDR cable tester [40]. The TDR cable tester (Triplett Test Equipment, Manchester, NH, USA) generates a pulse that propagates from the starting end of the probe. The arrival of the pulse at the terminal end of the probe is automatically identified by the system through the detection of a discontinuity point. These two points correspond to the propagation distance of the reflected electromagnetic waves, which is used to calculate the propagation time (Δ*t*), and subsequently, to determine the dielectric constant (*K_a_*) of the surrounding soil medium. The *K_a_* depends on the dielectric permittivity of the individual soil components (such as the solid particles, water, and air), as well as their volumetric fractions and geometric arrangement. Consequently, the volumetric water content (*θ_v_*) of the soil can be estimated through a calibration relationship between the *K_a_* and *θ_v_*. Such relationships enhance the convenience and accuracy of soil water content assessments based on TDR, making it an essential tool in soil science and engineering [41,42].

For the past decades, various *θ_v_*-*K_a_* models have been proposed. For example, Topp et al. [43] established a relationship between *θ_v_* and *K_a_* for soils (i.e., Equation (1)), finding that this relationship was largely unaffected by variations in soil bulk density, texture, salinity, and temperature. However, the applicability of this model is limited, as it is only valid for unfrozen soils and soils with low specific surface area.

(1) $$\theta_{v}=4.3\times 10^{-6}K_{a}^{3}-5.5\times 10^{-4}K_{a}^{2}+2.92\times 10^{-2}K_{a}-5.3\times 10^{-2}$$

Smith and Tice [44] investigated the relationship between *K_a_* and unfrozen water content based on NMR and TDR measurements on 25 different types of soils. Their results indicated that this relationship, shown in Equation (2), exhibits only a slight dependence on soil temperature or soil types. This finding suggests that the dielectric constant of soils can serve as a relatively stable and accurate indicator for estimating unfrozen water content under varying soil conditions.

(2) $$\theta_{v}=9.920\times 10^{-6}K_{a}^{3}-8.502\times 10^{-4}K_{a}^{2}+3.868\times 10^{-2}K_{a}-1.458\times 10^{-1}$$

#### 2.2.2. Frequency Domain Reflectometry (FDR)

Similar to TDR, FDR also measures water content based on the *K_a_* of the bulk soil sample. The primary distinction between the two methods is that FDR correlates *K_a_* with the frequency of electromagnetic waves propagating through the soil, which is why it is also referred to as capacitive technology [45,46]. This technique determines the *K_a_* by measuring the charging time of the soil sample acting as a capacitor. In FDR, the capacitor operates in conjunction with an oscillator to form a tuned circuit. By monitoring changes in the operating frequency, variations in the *K_a_* can be detected. In other words, FDR measures the frequency difference between the input and output electromagnetic waves ([47]. Ultimately, this frequency difference is used to calculate the *K_a_* of the soil, from which the soil water content can be derived. For the FDR sensor (i.e., EC-5, METER Group, WA, USA) used in the present study, according to the manufacturer’s operation manual, the *K_a_* and *θ_v_* can be determined by Equation (3 and 4, respectively).

(3) $$K_{a}=\frac{1}{(-1.10570\times 10^{-9})(RAW^{3})+(3.575\times 10^{-6})(RAW^{2})-(3.9557\times 10^{-9})(RAW)+1.53153}$$

(4) $$\theta_{v}=(8.5\times 10^{-4})(RAW)-0.48$$

where *RAW* is the output signal of the EC-5 moisture sensor when using a METER datalogger with 3-V excitation.

#### 2.2.3. Nuclear Magnetic Resonance (NMR)

As mentioned previously, the measuring principle of NMR is based on the spatial density of hydrogen atoms in a soil sample. Using a soil sample with a given water content, the calibration of the NMR signal intensity as a function of temperature under positive temperature conditions is called the paramagnetic regression line (Equation (5)). Subsequently, an NMR test on the soil sample is conducted at a subzero temperature *T_i_* to obtain the signal intensity *M*_0_(*T_i_*). It is assumed that the paramagnetic regression line is also applicable at subzero temperatures, which means that the signal intensity corresponding to the total water content is *y*(*T_i_*). Based on this, the unfrozen water content (*w_u_*) under the subzero temperature in question (*T_i_*) can be calculated by Equation (6) [48],

(5) y(T)=aT+b

(6) $$w_{u}=\frac{M_{o}(T_{i})}{y(T_{i})}w_{0}=\frac{M_{o}(T_{i})}{aT_{i}+b}w_{0}$$

where *y* represents the NMR signal intensity, *T* is the temperature (°C), *a* and *b* are fitting parameters, and *w*_0_ denotes the initial (total) water content (%) of the soil sample.

For porous media, such as soils, NMR can be used to quantify the pore size distribution of the soil by measuring the transverse relaxation time (T_2_) of hydrogen atoms. The relationship between T_2_ and the geometric morphology of the soil pores is typically expressed as

(7) $$\frac{1}{T_{2}}=\frac{\rho_{2}\times S}{V}$$

where *ρ*_2_ is the surface relaxation rate (μm/ms), which is a constant related to soil composition, *S* and *V* are the surface area (m^2^) and volume (m^3^) of the pores, respectively.

Equation (6) can be further transformed into

(8) $$R=\alpha \rho_{2}T_{2}$$

where *R* is the radius of the pore (μm), and *α* is a parameter related to the pore shape.

Finally, the following expression can be obtained:

(9) $$T_{2}=CR$$

where *C* is the pore radius conversion coefficient (ms/μm), which is a constant for specific types of soils [49].

### 2.3. Water Content Measurement of BAS

The original biochar particles were divided into two size ranges, coarse-grained (0.075~2 mm) and fine-grained (<0.075 mm), to facilitate the investigation of the impact of biochar particle size on water content measurement. The two sizes of biochar particles were incorporated into loess at four different dry mass ratios, that is, 0%, 3%, 6%, and 9%. A desired amount of distilled water was added to the dry loess–biochar mixture, which was then thoroughly mixed by hand. The wet mixture was stored in sealed plastic bags for at least 48 h to achieve uniform moisture distribution. For BAS water content measurement under room temperature condition, cylindrical specimens with a diameter of 5 cm and a height of 10 cm were used; while under subzero temperature measurement, the specimens assumed a diameter of 10 cm and a height of 10 cm. The void ratio of all the BAS specimens was controlled to a constant value of 0.8, with an initial water content of 15%. Those specimens were prepared by statically compacting the wet loess–biochar–distilled water mixture into five layers, each 2 cm thick. In total, seven types of BAS specimens were obtained for investigation. The BAS specimens were named by indicating the amount and particle size of biochar added. For example, BAS-3%F represents the BAS amended with fine-grained biochar, which has a biochar dosage of 3%; while BAS-9%C represents the BAS incorporating 9% coarse-grained biochar.

Different procedures were followed to measure the water content of BAS under room temperature and subzero temperature conditions, as described below.

**Figure 2:**
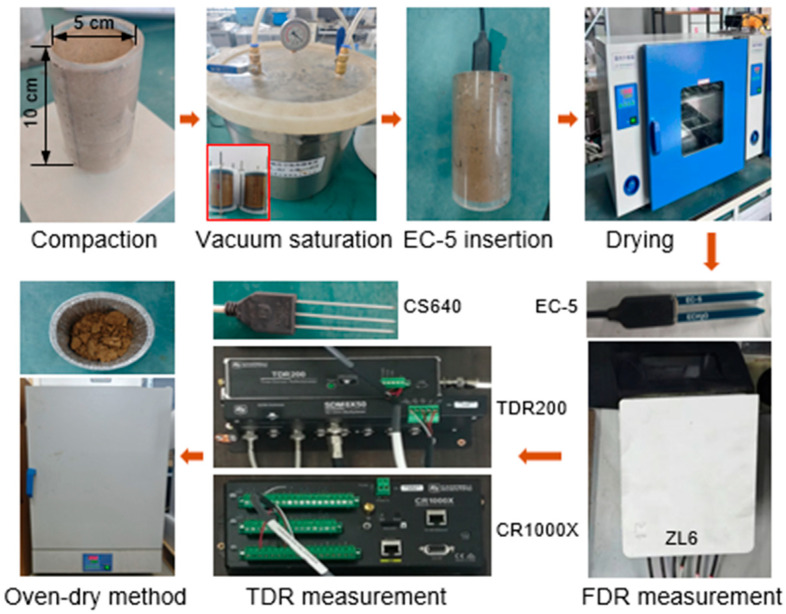
The process for water content measurement under room temperature.

**Figure 3:**
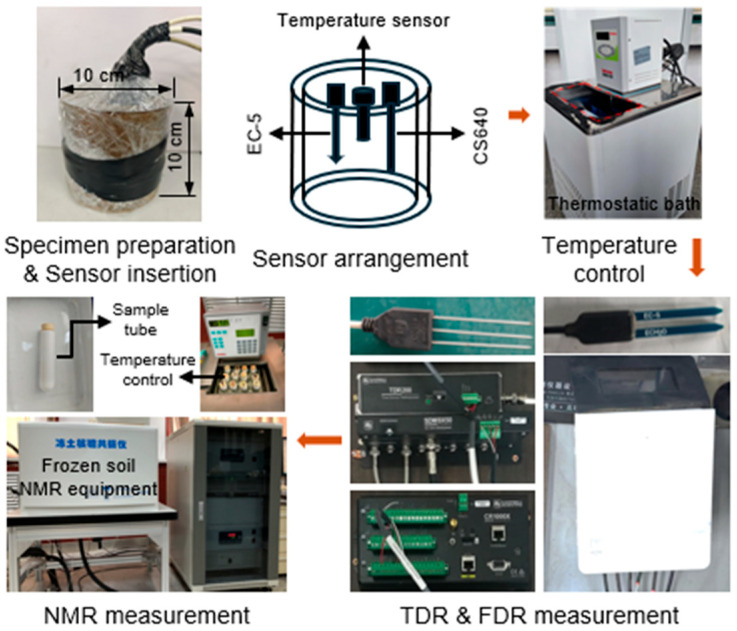
The process for water content measurement under subzero temperature.

(1) *Water content measurement of BAS under room temperature*. The experimental process is illustrated in Figure 2. First, all the BAS specimens were saturated using the vacuum saturation method. One EC-5 moisture sensor was then inserted into each of the specimens, which were subsequently placed in an oven with forced air circulation for drying to different moisture conditions. The temperature of the oven was controlled to a moderate value (i.e., 30 °C), preventing crack formation on the specimens due to fast temperature change. By doing so, the gravimetric water contents of the specimens were gradually adjusted to eight different target values: saturated state, 30%, 27%, 24%, 21%, 18%, 15%, and 12%. After that, the specimens were sealed with cling wrap and placed in a temperature-controlled chamber (approximately 20 °C) for 48 h to ensure uniform water and temperature distribution. Finally, the water contents of these specimens were measured by the EC-5 moisture sensor (Equation (3)), which was connected to a ZL-6 datalogger (METER Group, Pullman, WA, USA). The EC-5 was removed once the measurement was finished, and a CS640 sensor (Campbell Scientific, North Logan, UT, USA) was similarly inserted into the specimens for measurement using a TDR200 (Campbell Scientific, UT, USA) (Equation (1)), with data collected by a CR1000X datalogger (Campbell Scientific, UT, USA). After all measurements were completed, soil samples were taken from the specimens, and their actual gravimetric water contents were determined using the oven-dry method.
(2) *Water content measurement of BAS under subzero temperatures*. The experimental process is illustrated in Figure 3. Similar to the room temperature measurement scenario, the specimens were first subject to vacuum saturation treatment. Then, an EC-5, a CS640, and a temperature sensor were inserted into the specimen. To prevent water loss, the specimens with inserted sensors were sealed using cling wrap. Subsequently, they were placed in water tight plastic bags and positioned in a thermostatic bath, which served to freeze the specimens to different subzero temperatures. During the freezing process, the temperature of the thermostatic bath was adjusted according to the following steps: 0, −1, −2, −3, −5, −7, −10, −12, −15, −17, −20, −15, −10, −7, −5, −3, −2, −1, −0.7, −0.5, −0.3, and 0 °C. The specimens were maintained at least 12 h under each controlled temperature for equilibration. Once the equilibrium condition was achieved, the unfrozen water content of the specimens was determined by the EC-5 and CS640 sensors, based on Equation (3) and Equation (2), respectively.

In addition to the FDR and TDR measurements, at each subzero temperature, the unfrozen water content of the specimens was measured by using NMR (see Figure 3). The samples used for the NMR tests were from the cutting-ring specimens, with a height of 2 cm and a diameter of 6.18 cm. The initial water content of the compacted cutting-ring specimens was similarly controlled at 15%, with a void ratio of 0.8. After compaction, the specimens were saturated using the vacuum saturation method. Once saturation was complete, the specimens were cut into rectangular prisms approximately 1 × 1 × 3 cm (L × W × H) using a wire saw, ensuring that the mass of each soil sample was nearly equal. The trimmed samples were then placed into NMR sample holding tubes and equilibrated in thermostatic bath for 12 h before measurement.

## 3. Experimental Results and Analysis

### 3.1. Effects of Biochar on BAS Pore Structure

Using mercury intrusion test data to calibrate the NMR T_2_ spectrum allows for accurate determination of the pore radius conversion coefficient (*C*) for soil specimens. However, it is often not practically feasible to conduct mercury intrusion tests on all specimens. For specimens lacking such data, the conversion of T_2_ spectrum typically relies on the arithmetic mean of the *C* values obtained from multiple measured specimens or the development of statistical models [50]. Yang [51] combined the mercury intrusion and NMR results of BAS with varying biochar dosages (0%, 5%, 10%, 15%, and 20%) to derive the *C* values for these samples. Assuming this relationship is applicable to the BAS specimens in the present study, through interpolation based on biochar dosage, the *C* values for biochar dosages of 3%, 6%, and 9% were determined to be 18.20, 16.88, and 15.84, respectively.

By using Equation (10), the volume of pore water *V_i_* at a specific pore size (*R* in Equation (8)) can be calculated, thereby establishing a relationship with the NMR signal intensity [52]. To better compare the water content of soil samples with different dry densities, the normalized pore water volume, *V_n_* (i.e., pore water volume per gram of dry soil, cm^3^/g), can be calculated using Equation (11):

(10) $$V_{i}=\frac{A_{i}}{\sum A_{i}}\frac{m_{w}}{\rho_{w}}=\frac{A_{i}}{\sum A_{i}}\frac{m_{t}-m_{d}}{\rho_{w}}$$

(11) $$V_{n}=\frac{V_{i}}{m_{d}}=\frac{A_{i}}{\sum A_{i}}\frac{m_{t}-m_{d}}{\rho_{w}m_{d}}$$

where *A_i_* is the NMR signal intensity corresponding to a given T_2_ value, *m_w_* is the mass of soil pore water (g), *m_t_* is the total mass of the wet soil (g), *m_d_* is the dry soil mass (g), and *ρ_w_* is the water density (1.0 g/cm^3^).

**Figure 4:**
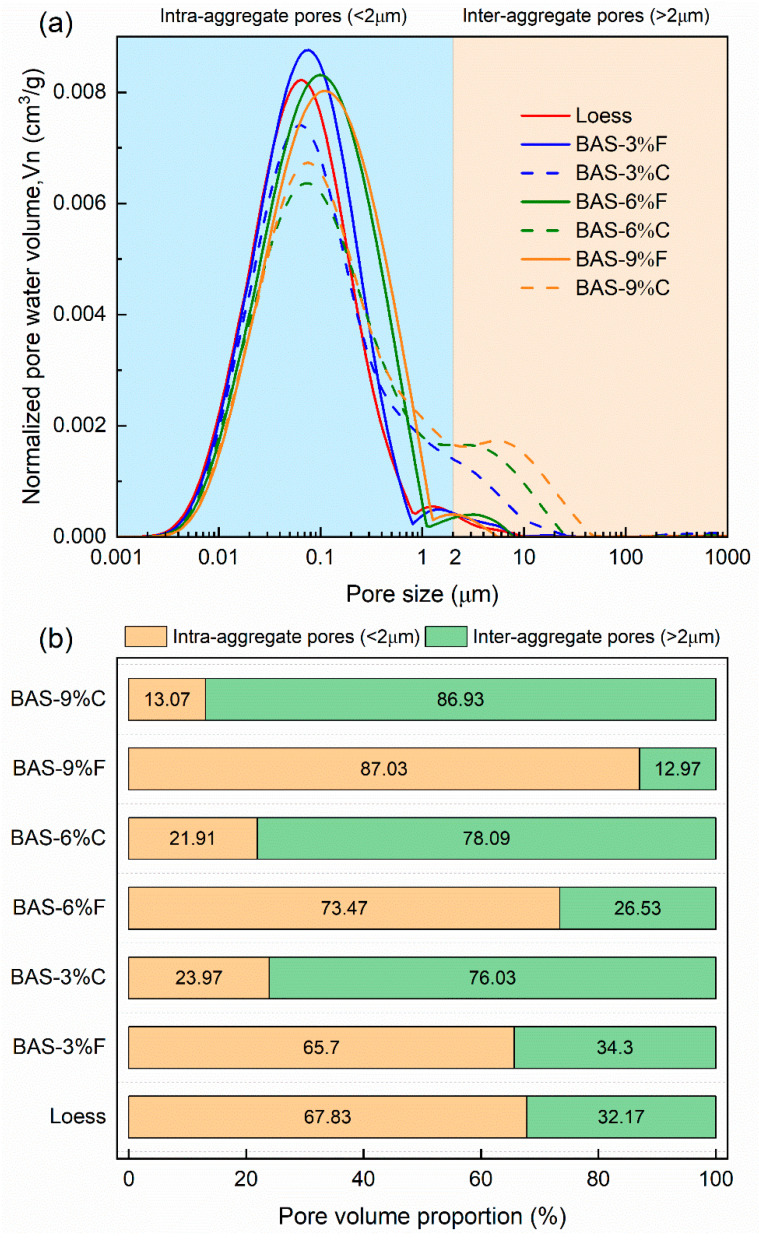
(**a**) Pore size distribution and (**b**) pore volume proportion of the BAS specimens.

Based on the above calculations, the pore size distribution curves of the seven types of BAS specimens were obtained, as shown in Figure 4a. It can be seen that the addition of biochar generally resulted in a bimodal pore structure. As the biochar dosage increases, the main peak of the curves gradually shifts to the righthand side. Furthermore, the height of the main peak for the BAS specimens with fine-grained biochar is much higher than that of the coarse-grained biochar-amended specimens. At the same time, the secondary peak for the BAS specimens with coarse-grained biochar shows a notable increase in height and shifts to the righthand side with increasing biochar dosage.

To better quantify the effects of biochar dosage and particle size on BAS pore structure, the pores were categorized into intra-aggregate (<2 μm) and inter-aggregate pores (>2 μm). Figure 4b displays the percentage of these two types of pores in the BAS specimens. The results indicate that the addition of coarse-grained biochar significantly increases the inter-aggregate pores while reducing the intra-aggregate pores, compared to the loess specimen (BAS-0%). Additionally, the amount of inter-aggregate pores positively correlated to the dosage of coarse-grained biochar. This is partly because coarse-grained biochar itself contains some pores larger than 2 μm [53,54]. In addition, due to its relatively rough surface, coarse-grained biochar is more likely to form larger pores when combined with loess particles to form aggregates. On the contrary, when fine-grained biochar is added, the inter-aggregate pores decrease while the intra-aggregate pores increase. This may be attributed to the smaller particle size of fine-grained biochar, which can fill the gaps between loess particles, transforming the originally larger pores into smaller ones. Such changes indicate that different particle sizes of biochar may have different influencing mechanisms on the pore structure of BAS.

### 3.2. Effects of Biochar on TDR Waveforms, BAS Electrical Conductivity, and Dielectric Constant

**Figure 5:**
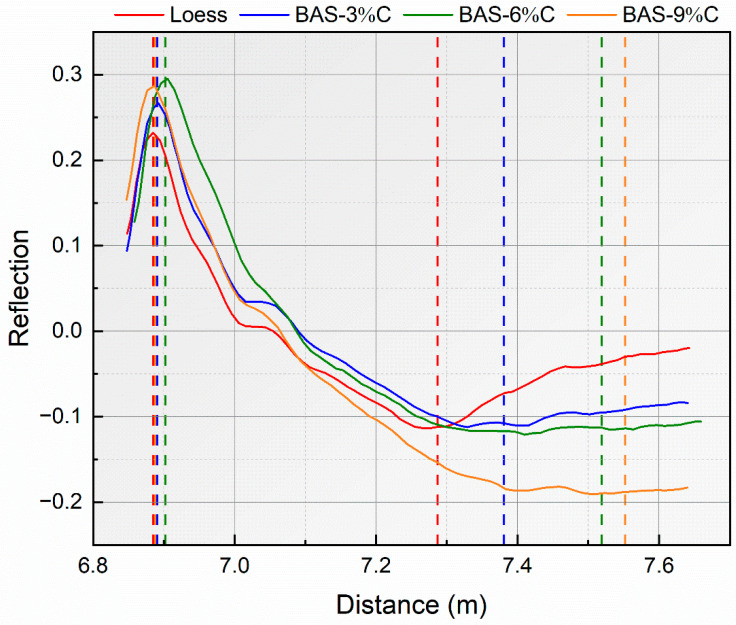
The TDR waveforms of the loess specimen, BAS-3%C, BAS-6%C, and BAS-9%C.

The TDR waveforms of BAS specimens with 0%, 3%, 6%, and 9% coarse-grained biochar are shown in Figure 5, with all the four specimens in a saturated state and tested under room-temperature conditions. It can be seen that the position of the second reflection point on the reflected wave increased from 7.287 m in the loess specimen to 7.552 m in BAS-9%C. In other words, the apparent length of the reflected wave increased from 0.402 m in loess to 0.663 m in BAS-9%C, showing a significant increase. Furthermore, as the biochar dosage increases, the propagation distance and time of the reflected wave in the BAS gradually extend. In addition, the intensity of the reflected signal at the second reflection point diminishes progressively, transitioning from a distinct inflection point to a smooth curve, with the increase in biochar dosage.

**Figure 6:**
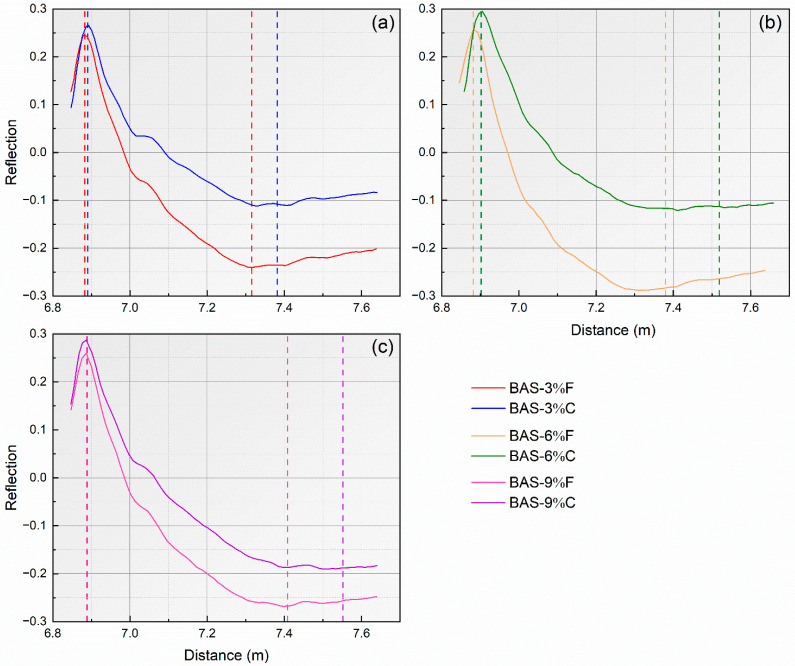
Comparison between the TDR waveforms of BAS specimens with (**a**) 3%, (**b**) 6%, and (**c**) 9% fine- and coarse-grained biochar.

The comparison between the TDR waveforms of coarse-grained and fine-grained biochar-amended loess with 3%, 6%, and 9% dosages are summarized in Figure 6. The results indicate that, at the same biochar dosage, the addition of coarse-grained biochar significantly increased both the propagation distance and time of the reflected wave, compared to the fine-grained biochar. Moreover, as the biochar dosages increased, the magnitude of the increase in the propagation distance of the reflected wave became more pronounced. For instance, the apparent length of the reflected wave of BAS-3%C was 0.058 m longer than that of BAS-3%F, while that of BAS-9%C was 0.143 m longer compared to BAS-9%F, indicating a clear upward trend. Furthermore, under the same biochar dosage, the coarse-grained biochar demonstrated a more significant effect on the dissipation of the reflected signal at the second reflection point. This phenomenon is consistent with previous research findings by Wanniarachchi et al. (2019) [32].

**Figure 7:**
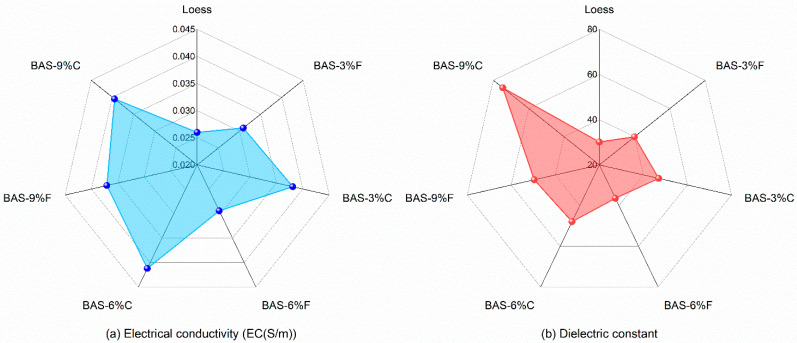
(**a**) Electrical conductivities and (**b**) dielectric constants of saturated BAS specimens under room temperature.

Figure 7 presents the electrical conductivity (EC) values and dielectric constants (*K_a_*) of the seven saturated BAS specimens measured by TDR under room-temperature condition. The results indicate that the incorporation of biochar significantly enhanced both the soil EC and *K_a_*. Specifically, when the loess was amended with 9% coarse-grained biochar, the EC increased from 0.026 S/m to 0.0395 S/m, while the *K_a_* t rose from 30.1 to 74.7. Furthermore, a positive correlation can be observed between the EC and the rate of biochar addition, with coarse-grained biochar exerting a more pronounced effect on the EC compared to fine-grained biochar. This trend is similarly reflected in the *K_a_* of the BAS specimens. These findings align with the observed impacts of biochar on TDR waveforms. The underlying mechanisms can be attributed to the high EC of biochar, which is primarily composed of carbon and contains a certain amount of surface ions. When biochar is introduced into the soil, it establishes a conductive network that facilitates charge transport. Moreover, the carbon skeleton of biochar acts as an effective medium for electron transfer, thereby enhancing the overall EC of the amended soil [55,56].

### 3.3. BAS Dielectric Constant Representation by a Dielectric Mixing Model

Roth et al. [57] proposed a comprehensive dielectric mixing model for soils, which treats soil constituents as distinct phases with independent dielectric properties and employs a weighted averaging approach to predict the dielectric constant of soils (*ε_mix_*). The model accounts for the dielectric response and volumetric fraction of each phase while describing the inter-phase interactions influencing the overall soil dielectric behavior. To extend this framework for biochar-amended soils in the present study, the effects of both biochar and ice formation were incorporated. The resulting dielectric mixing model for BAS can be expressed as

(12) $$\varepsilon_{mix}^{\alpha }=(\theta_{s}\varepsilon_{s}^{\alpha }+\theta_{w}\varepsilon_{w}^{\alpha }+\theta_{a}\varepsilon_{a}^{\alpha }+\theta_{b}\varepsilon_{b}^{\alpha }+\theta_{i}\varepsilon_{i}^{\alpha })$$

where *ε_s_*_,_ *ε_w_*, *ε_a_*, *ε_b_*_,_ and *ε_i_* represent the dielectric constants of solid particles, liquid water, air, biochar particles, and ice, respectively (*ε_w_* = 80, *ε_a_* = 1, and *ε_i_* = 3.2). *α* is a geometry-dependent parameter related to the spatial arrangement of solid particles (typically assumed as 0.5 for isotropic media), and *θ_s_*, *θ_w_*, *θ_a_*, *θ_b_* and *θ_i_* denote the volumetric fractions of each phase.

**Figure 8:**
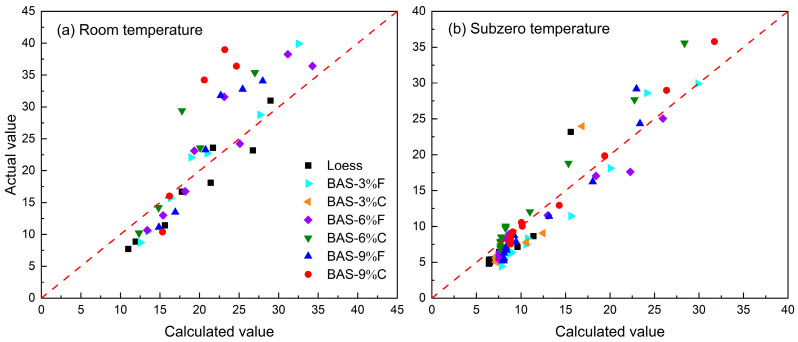
Comparison between the actual and calculated dielectric constants of BAS specimens under (**a**) room temperature and (**b**) subzero temperature.

The methodology consists of three sequential steps: (1) The dielectric constant of loess particles was back-calculated based on that of the saturated loess specimen and the volumetric fractions of loess particles and water. (2) The dielectric constant of biochar particles was obtained from a compacted biochar specimen based on TDR measurements and known volumetric fractions of biochar particles, water, and air. (3) These back-calculated values (the dielectric constants of loess and biochar particles were 6.85 and 35.11, respectively) were then incorporated into the mixing model to determine the theoretical dielectric constant of the BAS specimens under both the room-temperature and subzero-temperature conditions. As shown in Figure 8, the model’s calculations are in good agreement with the experimentally determined dielectric constants of the BAS specimens. Although some discrepancies were observed within a certain range, these can be attributed to variations in the α parameter caused by differences in mineral composition and particle spatial arrangement. Nevertheless, the overall error remains small, demonstrating the model’s capability to effectively predict the dielectric constant of BAS specimens, with the α parameter of 0.5 as a good approximation.

### 3.4. Comparison of Measured Water Contents with Actual Values

**Figure 9:**
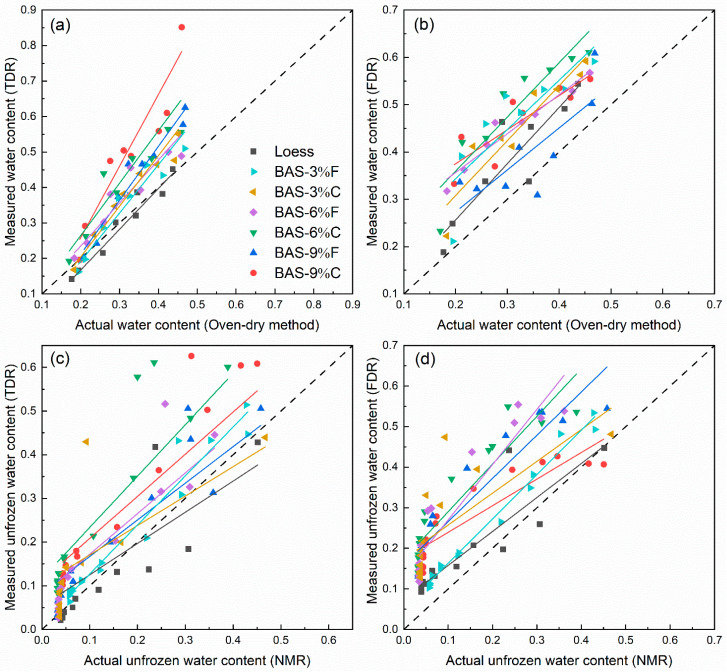
Comparison between the measured and actual (unfrozen) water contents: (**a**,**c**) TDR, and (**b**,**d**) FDR.

The water contents of BAS specimens measured by TDR and FDR under room temperature were compared with the actual water contents determined by the oven-dry method, as shown in Figure 9a,b. Similarly, the comparison between the unfrozen water contents measured by TDR and FDR under subzero temperature conditions with those obtained from NMR are summarized in Figure 9c,d. It can be seen that for the loess specimen, TDR tended to underestimate its water content under both room- and subzero-temperature conditions, while FDR overestimated the water content of the loess specimen at room temperature, but under subzero-temperature conditions, it underestimated and overestimated the unfrozen water content when the water content was high and low, respectively. In the case of BAS, both at room and subzero temperatures, the fitted lines for both the TDR and FDR methods lie above the 1:1 line, indicating that both methods overestimated the water content of the BAS specimens. Furthermore, the fitted line for the loess specimen has a slope closest to 1, and as the biochar dosage increases, the slope of the fitted line gradually increases, deviating from the reference line. Notably, for the BAS-9%C, the slope of the fitted line at room temperature even exceeds 2. These results suggest that the incorporation of biochar significantly affected the TDR and FDR measurements, highlighting the need for caution when using these methods to assess BAS water contents.

**Figure 10:**
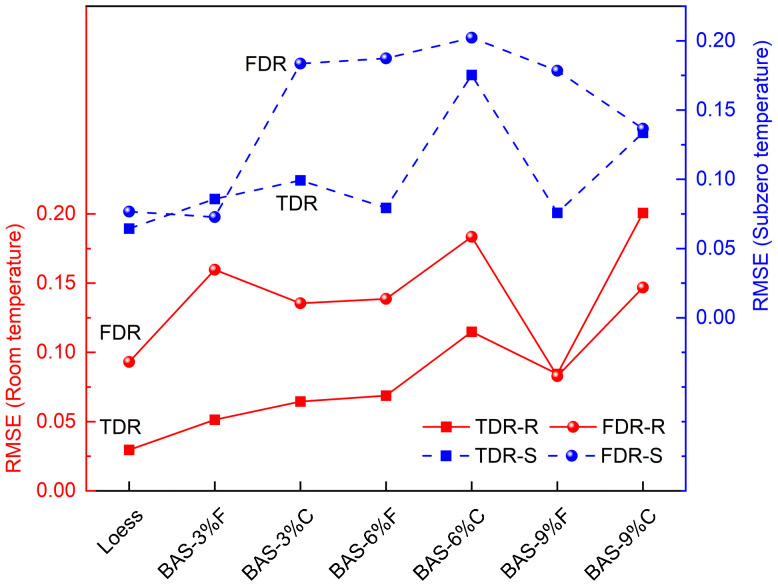
The RMSE between measured and actual (unfrozen) water contents under room temperature and subzero temperature (TDR-R: RMSE of TDR at room temperature; FDR-R: RMSE of FDR at room temperature; TDR-S: RMSE of TDR at subzero temperature; FDR-R: RMSE of FDR at subzero temperature).

To analyze the measurement errors more accurately, the root mean square errors (RMSE) between the TDR- and FDR-measured water contents and their actual values under both room- and subzero-temperature conditions were calculated. As shown in Figure 10, the RMSE for both methods when measuring the water content of the loess specimen was lower than that for the amended specimens, and the RMSE for TDR was generally lower than that for FDR. This again indicates that the addition of biochar affected the accuracy of the water content measurements by both methods, with FDR exhibiting significantly higher errors compared to TDR. Furthermore, as the biochar dosage increased, the measurement errors gradually increased. Under the same biochar dosage conditions, the impact of coarse-grained biochar on the measurement accuracy of both methods was generally more pronounced. This finding is consistent with the trend observed in the slopes of the fitted lines, which increase as the biochar dosage rises. Therefore, investigating the isolated effect of biochar on soil water measurement is of significant scientific necessity. Such studies not only contribute valuable insights to the field of water measurement in BAS but also help elucidate the independent role of biochar, which can serve as a critical parameter for multifactorial coupling models (e.g., salinity interactions) [58].

### 3.5. Calibration for BAS Water Content Measurement

**Figure 11:**
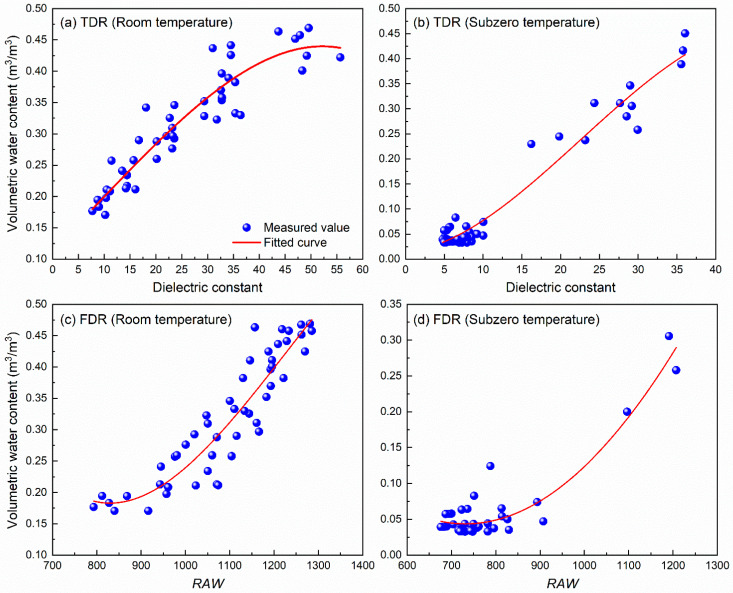
Calibration results for BAS water content: (**a**,**b**) TDR and (**c**,**d**) FDR.

Soil water content calibration is essential for establishing accurate quantitative relationships between the physical signals measured by moisture sensors (such as voltage, frequency, or dielectric constant) and the actual water content of the soil. In this study, such calibration relationships were established by fitting the *K_a_* measured by TDR and the *RAW* values (see Equation (3)) obtained from FDR (EC-5 sensor) with the actual water content determined under both the room and subzero temperature conditions, for the seven types of BAS specimens, as shown in Figure 11. The resulting fitting equations and parameters, as well as the coefficient of determination (R^2^) are summarized in Table 2. It can be concluded that the best-fit calibration curves are generally able to well describe the relationship between BAS water content and its *K_a_* or the *RAW* value, regardless of the biochar dosage or particle size. Although this may compromise the accuracy of water content determination for a specific BAS specimen, it is easy to use since one single calibration equation is obtained for different types of BAS specimens, by using TDR or FDR, under either room- or subzero-temperature conditions. However, the current model may not be directly applicable to complex natural soils due to their inherent heterogeneity. Future studies should incorporate soil texture correction factors or employ multivariate regression models approaches to optimize the predictive equations.

**Figure 12:**
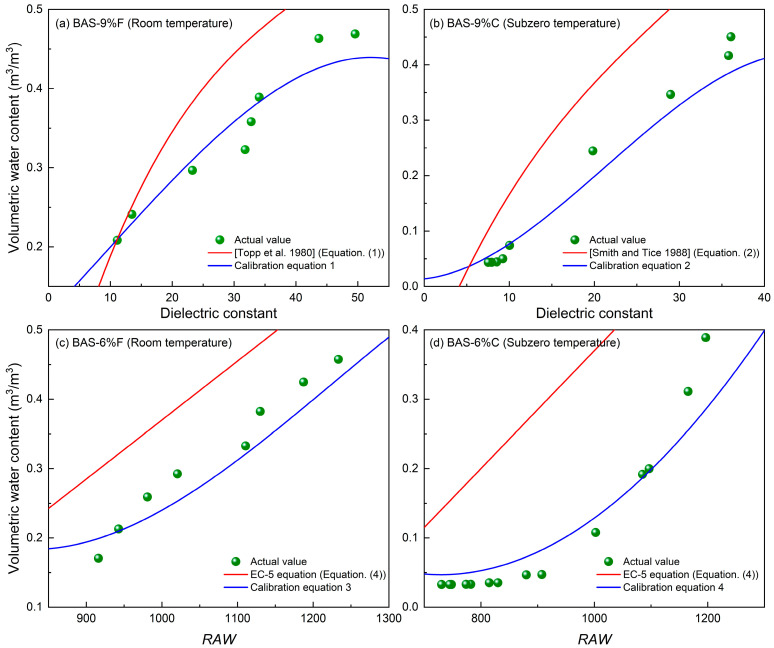
Comparison of actual water contents with those determined by empirical and calibration equations for (**a**) BAS-9%F, (**b**) BAS-9%C, (**c**) BAS-6%F, and (**d**) BAS-6%C.

As an example, a comparison between the actual water contents of four BAS specimens and the results based on the empirical formulas (i.e., Topp et al. [43] equation, Smith and Tice [44] equation, and EC-5 equation) is illustrated in Figure 12. Also shown are the results determined by using the above calibration equations. Overall, the calibration equations developed in this study demonstrate superior performance in converting the dielectric constant or *RAW* value into water content, exhibiting good agreement with the actual values. In contrast, significant discrepancies are observed between the water contents derived from the Topp et al. [43], Smith and Tice [44], and EC-5 equations and their actual counterparts. As a result, these empirical relationships that were developed for typical non-biochar-amended soils cannot be directly used to determine the water content of BAS without calibration. However, the current model may not be directly applicable to complex natural soils due to their inherent heterogeneity. Future studies should incorporate soil texture correction factors or employ multivariate regression models approaches to optimize the predictive equations.

## 4. Discussion

The measurement accuracy of dielectric sensors is influenced by multiple factors. Firstly, different types of soils possess distinct dielectric constants, and the solid components within the soil (such as minerals) can also affect the measurements [59]). Secondly, the salts in the soil significantly affect the measurement results. High salinity can alter soil electrical conductivity, which in turn affects the propagation speed of electromagnetic waves, leading to errors in water content measurements [60]. Additionally, the density and porosity of the soil can also influence the performance of dielectric sensors. Soils with higher porosity may exhibit a different relationship between water content and dielectric constant compared to denser soils [61]. Finally, the addition of conductive materials, such as biochar, can modify the electrical properties of the soil, thereby affecting the reading and measurement accuracy of dielectric sensors. To enhance the precision of dielectric sensors, it is essential to calibrate or account for these factors during measurements [62,63].

**Figure 13:**
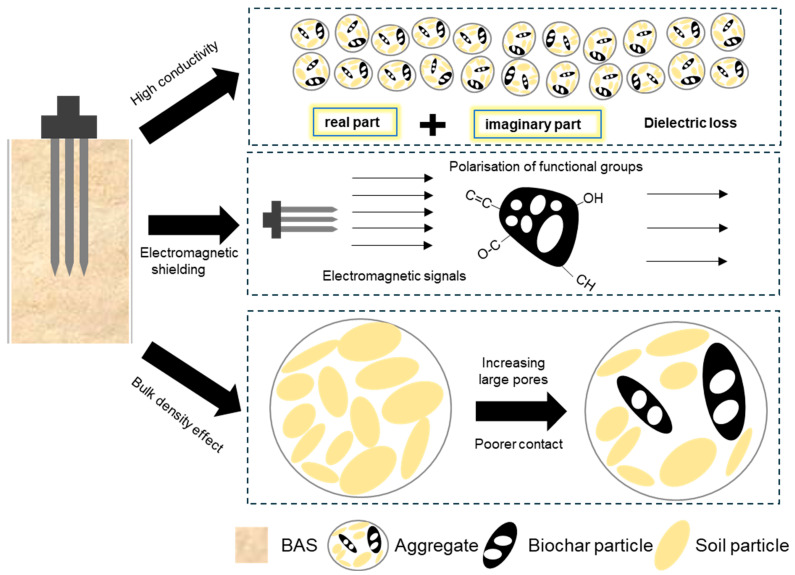
Influencing mechanisms of biochar on TDR and FDR water content measurement.

Based on the analysis of biochar properties and factors influencing dielectric sensor performance, the overall impact mechanisms of biochar on TDR and FDR water content measurement can be categorized into three types: high conductivity, electromagnetic shielding, and bulk density effects. The influence mechanisms are schematically illustrated in Figure 13. The soil dielectric constant is a complex variable, with its real part representing the energy stored in the dielectric medium, while the imaginary part indicates dielectric loss and conductive loss. Generally, at TDR frequencies, the imaginary component is assumed to be negligible, meaning that the dielectric constant measured by TDR primarily reflects its real part. However, when measuring conductive materials, the influence of the imaginary component cannot be overlooked [64]. Biochar, as a highly conductive material [56], significantly enhances soil’s electrical conductivity when incorporated, as seen in Figure 7a. If the imaginary part is ignored during measurements, it may lead to an overestimation of the dielectric constant, resulting in exaggerated calculations of (unfrozen) water content [65]. As the proportion of biochar increases, the influence of the imaginary part will increase significantly. This phenomenon aligns with our observation that the measurement errors in TDR increase with the rising biochar dosage. Therefore, considering the imaginary part of the dielectric constant is crucial for improving measurement accuracy in BAS water content monitoring.

Biochar is well known not only as a conductive material but also for its ability to provide electromagnetic shielding effects [66]. Its carbon-based structure enables the formation of conductive pathways under the influence of an electromagnetic field. When electromagnetic waves pass through biochar, a portion of the electromagnetic energy is absorbed, reflected, or scattered by these conductive pathways. Particularly under high-frequency electromagnetic wave conditions, the conductive pathways within biochar can effectively reduce the transmission of electromagnetic waves, thereby serving a shielding function [67]. Moreover, the porous structure of biochar significantly increases its surface area, providing more interfaces for interaction with electromagnetic waves. During the propagation of electromagnetic waves, longer-wavelength signals may scatter or reflect within biochar pores, reducing the penetration depth of the electromagnetic signal [68]. Additionally, the polar functional groups on the surface of biochar undergo polarization in the presence of an electromagnetic field, where the molecules or clusters of these polar groups rearrange, leading to the absorption of electromagnetic energy [69]. This polarization effect results in the absorption of electromagnetic waves at the surface of biochar, converting them into thermal energy and further diminishing the electromagnetic energy transmitted into the interior of biochar, thereby enhancing its shielding effectiveness [70,71]). Research conducted by Wang and Hung [72]) indicated that the charcoal produced from six types of wood significantly influenced electromagnetic shielding against electric fields. Furthermore, Khushnood et al. [73] demonstrated that adding 0.5% biochar to ordinary cement composites significantly enhanced their electromagnetic radiation-shielding effectiveness. These findings underscore the substantial potential of biochar in improving the electromagnetic shielding properties of materials, warranting further exploration and application in soil remediation and environmental protection.

The accuracy of dielectric sensors in measuring water content is influenced by the soil bulk density. This factor reflects the degree of contact between the sensor and soil matrix [74]. As shown in Figure 4, the addition of biochar significantly affects the pore structure of the soil. For example, adding coarse-grained biochar resulted in an increase in larger-size pores within the BAS. This change may lead to poorer contact between moisture sensor and soil matrix, as a greater number of pores implies a reduction in the contact area between the two, thereby introducing measurement errors. In contrast, when fine-grained biochar is added, the pore structure of the BAS transitions from inter-aggregate pores to intra-aggregate pores as biochar dosage increases. This transformation may enhance the contact between the sensor and soil matrix, thereby partially reducing measurement errors. Consequently, the addition of fine-grained biochar may result in smaller errors. This finding underscores the significant impact of biochar particle size on the performance of moisture sensors, suggesting that the type and dosage of biochar should be considered when monitoring soil water dynamics.

There are also several limitations of the present study that warrant consideration. First, the investigation was conducted using only a single soil type, which may limit the generalizability of the findings to other soil types. Second, the biochar feedstock employed in the experiments was restricted to a single source, potentially constraining the comprehensive evaluation of different biochar types on soil properties. Finally, significant discrepancies exist between controlled laboratory conditions and actual field environments, which may affect the extrapolation of experimental results to practical applications. To address these limitations, future studies should systematically examine the combined effects of various soil textures and biochar feedstocks on soil physicochemical properties. In addition, the singular effect of biochar was considered, while the combined influence of factors such as salts, mineral components, and others should also be taken into account. Additionally, the development of multivariate models could enhance predictive accuracy by accounting for soil heterogeneity and environmental variables. Furthermore, integrating long-term field monitoring with laboratory experiments would improve model reliability and applicability, enabling a more dynamic assessment of biochar performance under diverse environmental conditions and its potential ecological implications. Given the adsorption capacity of biochar and clay for soil contaminants ([75], and the applicability of TDR and FDR techniques for pollutant measurement, future studies should develop contaminant-specific predictive models (e.g., for heavy metals and organic pollutants) [76,77]. Such models would enhance both wastewater treatment efficiency and the accuracy of pollutant concentration quantification, representing a critical direction for further research.

## 5. Summary

The present study investigated the effects of corn straw biochar dosages and particle size on the microstructure of loess, as well as their impact on the water content measurement accuracy of BAS using TDR and FDR techniques. The results indicate that the addition of coarse-grained biochar significantly increases the inter-aggregate pores of the soil while reducing the intra-aggregate pores. As biochar dosage increases, the proportion of inter-aggregate pores continues to increase. In contrast, incorporating fine-grained biochar leads to a decrease in inter-aggregate pores and an increase in intra-aggregate pores. Regarding TDR waveforms, coarse-grained biochar has a more pronounced effect on the waveforms compared to fine-grained biochar, and this effect intensifies with increasing biochar dosage. Moreover, biochar can significantly increase the electrical conductivity and dielectric constant of the soil, and the increase is more pronounced for coarse-grained biochar. These align with the trends observed in the water content measurement by using TDR and FDR, indicating that coarse-grained biochar has a greater impact on the measurement accuracy of both methods, with measurement errors significantly increasing as biochar dosage rises.

The influence of biochar addition and ice formation were incorporated into a classic dielectric mixing model, resulting in the development of a new model that is applicable to BAS under both room- and subzero-temperature conditions. The comparison between the measured dielectric constants of BAS and those calculated by the dielectric mixing model revealed a high degree of agreement. Additionally, calibration equations for accurately determining the water content of BAS specimens were established for the TDR and FDR sensors, under both room temperature and subzero temperatures. When compared with empirical formulas that were developed for typical non-biochar-amended soils, the calibration equations demonstrated superior accuracy in predicting the water content of BAS. However, the present study is limited to Lanzhou loess and four specific biochar dosages (0%, 3%, 6%, and 9%). Therefore, further research is needed to explore more soil types and biochar application rates for better elucidating the underlying influencing principles. This will provide a more comprehensive theoretical foundation and practical guidance for future soil improvement with biochar and BAS water content measurement.

## Figures and Tables

**Table 1:** Basic properties of loess and biochar.

**Loess**	**G_s_**		**LL** **(%)**	**PL** **(%)**	**PI** **(%)**	**OMC** **(%)**	**MDD** **(g/cm^3^)**	**USCS**	**pH**
	2.73		26.2	20.2	6.0	15.7	1.798	Silty Clay	7.80
**Biochar**	**G_s_**		**Ash content (%)**		**pH**	**Surface ion content (mg/L)**			
						K^+^	Ca^2+^	Cl	SO_4_^2^
	F	1.84	41.33		8.81	28.45	0.45	11.49	2.19
	C	1.58							

Note: Gs: specific gravity; LL: liquid limit; PL: plastic limit; PI: plasticity index; OMC: optimum moisture content; MDD: maximum dry density; USCS: Unified Soil Classification System [39]; F and C: fine- and coarse-grained biochar, respectively.

**Table 2:** Calibration results for BAS water content.

TDR	Calibration Equation *θ_v_* = a × (*K_a_*)^3^ + b × (*K_a_*)^2^ + c × *K_a_* + d					
	No.	a (×10^−6^)	b (×10^−4^)	c (×10^−3^)	d (×10^−2^)	R^2^
Room temperature	1	−1.56	−0.432	8.21	11.45	0.88
Subzero temperature	2	−8.16	5.52	1.64	1.37	0.96
**FDR**	**Calibration Equation** ***θ_v_*** **= a × (*RAW*)^3^** **+ b ×** **(*RAW*)^2^** **+ c × *RAW* + d**					
	**No.**	**a (×10^−10^)**	**b (×10^−6^)**	**c (×10^−2^)**	**d**	**R^2^**
Room temperature	3	−19.0	7.01	−7.71	2.839	0.84
Subzero temperature	4	−1.05	1.37	−1.83	0.694	0.89

## Data Availability

The data that support the findings of this study are available upon reasonable request.
